# Vascular-specific genome editing enhances low-phosphate tolerance in rice

**DOI:** 10.1093/plcell/koag203

**Published:** 2026-07-02

**Authors:** Ju-Chen Chia

**Affiliations:** Assistant Features Editor, The Plant Cell, American Society of Plant Biologists; Plant Biology Section, School of Integrative Plant Science, Cornell University, Ithaca, NY 14853, United States

Phosphorus (P) is an essential macronutrient for plant growth and development. Orthophosphate (Pi), the bioavailable form of phosphorus in soil, exhibits low solubility and mobility, which frequently causes phosphorus limitation in plants (Lambers 2022). Due to its low mobility in soil, only 15% to 25% of applied phosphate fertilizer is typically utilized by crops, whereas the remainder becomes unavailable in the soil or is lost to the environment, negatively affecting surrounding ecosystems. Therefore, improving phosphate-use efficiency remains a major objective for sustainable crop production.

To cope with Pi limitation, plants activate the phosphate starvation response (PSR) that enhances Pi acquisition and redistribution (Yang et al. 2024). At the transcriptional level, PSR is regulated by phosphate-sensing SPX (SYG1/PHO81/XPR1)-domain proteins, which repress the activity of the master transcription factor PHOSPHATE STARVATION RESPONSE2 (PHR2), involved in regulating low-Pi–responsive genes (Wang et al. 2014). In parallel, the E2 ubiquitin-conjugating enzyme PHOSPHATE 2 (PHO2) negatively regulates PSR at the post-translational level by promoting the turnover of phosphate transporters. In rice (*Oryza sativa*), OsPHO2 promotes the degradation of a Pi efflux transporter PHOSPHATE1;2 (OsPHO1;2), required for Pi transfer from root to shoot and Pi remobilization during grain filling (Wang et al. 2020; Ma et al. 2021).

Because SPX proteins and PHO2 act as negative regulators of PSR, loss-of-function mutations in these genes can enhance Pi uptake and translocation. However, constitutive disruption of these repressors often results in Pi overaccumulation and aberrant PSR activation (Hu et al. 2011; Wang et al. 2014). To overcome these limitations, **Guangbo Wei and colleagues (Wei et al. 2026)** developed a vascular-specific CRISPR/Cas9 strategy that would restrict gene disruption to tissues central to Pi translocation and PSR signaling.

To drive vascular-specific CRISPR/Cas9-mediated gene editing in rice, the authors selected the promoters of *OsPHO1;2* (*P_PHO1;2_*) and *OsSHR1* (*P_SHR1_*), which are specifically active in vascular tissues in both roots and shoots regardless of Pi status. Two gRNAs targeting *OsPHO2* were introduced into the vascular-specific CRISPR constructs to generate transgenic lines in rice cultivar “Nipponbare”, including *P_PHO1;2_-Cas9;gRNA-anti-OsPHO2* (*P_PHO1;2:pho2_*) and *P_SHR1_-Cas9;gRNA-anti-OsPHO2* (*P_SHR1;2:pho2_*). In parallel, the authors generated a whole-plant *OsPHO2* null mutant (*pho2*) using a ubiquitin promoter-driven CRISPR/Cas9 system.

The vascular-specific CRISPR/Cas9 system efficiently disrupted *OsPHO2* specifically in the vasculature and maintained editing activity across multiple generations. In contrast to the null *pho2* mutant, which hyperaccumulated Pi and developed leaf necrosis, the *P_PHO1;2:pho2_* and *P_SHR1;2:pho2_* plants exhibited only moderately increased Pi concentrations and leaf-to-root Pi ratios while maintaining normal growth under hydroponic conditions. These findings illustrate that vascular-specific *OsPHO2* knockout enhances Pi translocation and accumulation without compromising whole-plant Pi homeostasis. The agronomic benefits of this strategy became evident in low-Pi field trials. Compared with Nipponbare, the vascular-specific *pho2* lines produced significantly high number of tillers per plant, whereas the null *pho2* mutant displayed dwarfism and significantly reduced yield.

To investigate the molecular basis underlying the improved low-Pi tolerance, the authors compared the root transcriptomes of vascular-specific *pho2* lines, the null *pho2* mutant, and Nipponbare grown under Pi-sufficient and Pi-deficient conditions. The data revealed that vascular-specific *OsPHO2* knockout moderately enhanced the expression of *OsPHO2*-repressed, vascular-localized phosphate starvation-induced genes in response to Pi deficiency. Notably, the overall PSR signaling under low-Pi conditions remained largely comparable with that of Nipponbare. In contrast, the null *pho2* mutant exhibited extensive changes in PSR, metabolic pathways, and defense-related responses under Pi deficiency. These findings suggest that knocking out *PHO2* in vascular tissues prevents the global transcriptional reprogramming associated with complete *PHO2* loss.

The authors also obtained similar results by knocking out *OsPHO2* or *OsSPX1/2* in the Zhonghua11 background using this vascular-specific CRISPR system, demonstrating the robustness of this approach across rice cultivars. These plants exhibited enhanced low-Pi tolerance and agronomic performance in Pi-deficient paddies while maintaining whole-plant Pi homeostasis.

In summary, Wei et al. developed a vascular-specific CRISPR/Cas9 knockout system that improves low-Pi tolerance while maintaining the balance between plant growth and phosphate starvation responses (Fig. 1). An important next step will be to translate this proof-of-concept strategy into practical crop breeding applications. More broadly, this work highlights an important role for vascular tissues in the spatial regulation of PSR and opens new avenues for investigating vascular-specific PSR signaling networks in several crops.

**Figure 1:**
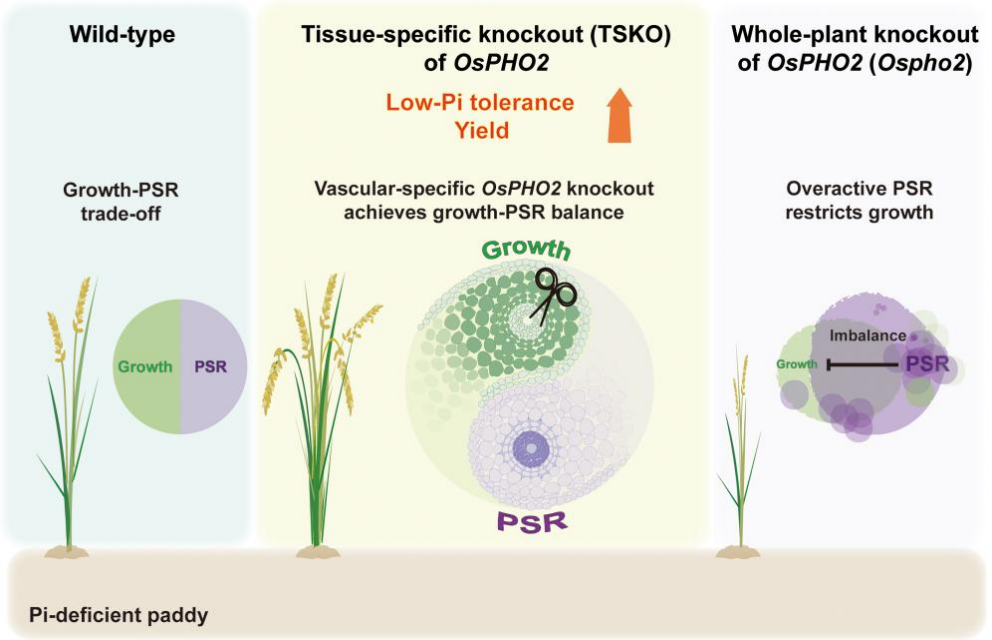
Vascular-specific knockout of *OsPHO2* balances PSR and growth under Pi-limiting conditions, thereby enhancing low-Pi tolerance and yield in rice. Adapted from Wei et al. (2026), Fig. 7.

## Recent related articles in *The Plant Cell:*

Zhang et al (2024) unveiled a distinctive post-translational regulatory mechanism in Pi signaling by which brassinosteroids (BRs) promote Pi acquisition. The authors demonstrated that GSK2, the key regulator of BR signaling, phosphorylates OsPHR2 at a specific serine (S) residue, S269, which suppresses OsPHR2 binding to the promoters of Pi starvation-induced genes and ultimately leads to reduced Pi uptake and accumulation in rice plants.

Hu et al. (2024) identified the Myb73-GDPD2-GA2ox1 regulatory module as a major determinant of phosphate deficiency tolerance in soybean. They demonstrated that GmMyb73 negatively regulates GmGDPD2 expression, whereas GmGDPD2 interacts with GmGA2ox1 to promote root development, phosphate acquisition, and phosphate-use efficiency under low-phosphate conditions.

Lin et al. (2025) showed that SlPHR3 and SlPHR4 act as central regulators of Pi homeostasis and phosphate starvation responses in tomato. The authors further identified SlGRAS47, SlBHLH48, and SlMYB28 as novel downstream regulators of this pathway.

Yang et al. (2025) revealed that phosphate starvation induces PHR1-dependent sphingolipid remodeling in Arabidopsis through the direct regulation of nonspecific phospholipase C4 (NPC4) and glucosylceramide synthase (GCS).

## Data Availability

No new data were generated or analyzed in support of this research.
